# Autoimmunity in Long Covid and POTS

**DOI:** 10.1093/oxfimm/iqad002

**Published:** 2023-03-08

**Authors:** Fatema-Zahra El-Rhermoul, Artur Fedorowski, Philip Eardley, Patricia Taraborrelli, Dimitrios Panagopoulos, Richard Sutton, Phang Boon Lim, Melanie Dani

**Affiliations:** Department of Allergy and Clinical Immunology, Guy’s and St Thomas’ NHS Foundation Trust, London SE1 9RT, UK; Department of Cardiology, Karolinska University Hospital and Karolinska Institute, Stockholm 171 77, Sweden; Imperial Syncope Unit, Imperial College Healthcare NHS Trust, London W12 0HS, UK; Imperial Syncope Unit, Imperial College Healthcare NHS Trust, London W12 0HS, UK; Imperial Syncope Unit, Imperial College Healthcare NHS Trust, London W12 0HS, UK; National Heart and Lung Institute, Imperial College London, London SW3 6LY, UK; Imperial Syncope Unit, Imperial College Healthcare NHS Trust, London W12 0HS, UK; Imperial Syncope Unit, Imperial College Healthcare NHS Trust, London W12 0HS, UK; Cutrale Perioperative and Ageing Group, Department of Bioengineering, Imperial College London, London W12 0BZ, UK

**Keywords:** autoimmunity, POTS, Long Covid, autoantibody, autoimmune

## Abstract

Orthostatic intolerance and other autonomic dysfunction syndromes are emerging as distinct symptom clusters in Long Covid. Often accompanying these are common, multi-system constitutional features such as fatigue, malaise and skin rashes which can signify generalized immune dysregulation. At the same time, multiple autoantibodies are identified in both Covid-related autonomic disorders and non-Covid autonomic disorders, implying a possible underlying autoimmune pathology. The lack of specificity of these findings precludes direct interpretations of cause and association, but their prevalence with its supporting evidence is compelling.

## The autonomic nervous system and immune systems in acute and Long Covid—a complex relationship

The immune and autonomic nervous systems have a complex, reciprocal relationship which affects peripheral immune responses, particularly related to cytokines [1, 2]. Cells of the immune system express adrenergic and nicotinic receptors and can also release catecholamines [3, 4]. This relationship is evident in both acute Covid infection and later sequelae. The well-established cytokine release syndrome in acute Covid infection, clinically manifesting as acute respiratory distress syndrome (ARDS), is characterized by sympathetically mediated pro-inflammatory cytokine cascade (driven by IL-6) [5, 6]. It has been hypothesized that the chronically elevated sympatho-activation in existing conditions such as hypertension, heart disease and metabolic syndrome drives exaggerated sympathetic responses and poor outcomes [7]. The spike protein of the SARS-CoV-2 virus binds the angiotensin converting enzyme 2 (ACEII) receptor, resulting in increased angiotensin 2 activity and vasoconstriction [7].

On the other hand, the parasympathetically mediated cholinergic anti-inflammatory reflex regulates this sympathetically related inflammation by inhibiting cytokine production from macrophages thus reducing TNFα production [7–9]. This vagally mediated immune modulation is illustrated by a recent randomized controlled trial which found that transcutaneous auricular (non-invasive) vagal nerve stimulation reduces acute inflammation markers such as C-reactive protein in hospitalized Covid patients [10].

Further evidence of neuroimmune interactions is the finding that autonomic system autoantibodies which are present in both POTS and Long Covid [G-protein coupled receptor (GPCR) antibodies] [11, 12] are also present in acute Covid infection and correlate with disease severity [13].

The relationship between Long Covid, the autonomic nervous system and associated autoimmunity still needs full evaluation but unifying findings are emerging. In patients infected with Covid, a range of autoantibodies is seen against immune proteins such as cytokines, complement and cell surface proteins [14]. These autoantibodies may alter the immune response to acute infection, with correlations with inflammatory biomarkers such as C-reactive protein [14]. Anti-cardiac antibodies have also been identified in the acute severe phase of Covid infection, but without correlation with clinical outcome [15].

Patients with Long Covid have upregulated pro-inflammatory cytokines (IFNα, TNFα, IL6, IL17α, IL1β, IL14) [16] which persist for up to 9–12 months following infection [17] and are associated with symptoms at 12 months [18]. Autoantibodies are identified in both Covid-related autonomic disorders and non-Covid autonomic disorders, implying a possible underlying autoimmune pathology. The lack of specificity of these findings precludes direct interpretations of cause and association, but prevalence with its supporting evidence is compelling.

In this review, we discuss the role of the autonomic nervous and immune systems in Covid and Long Covid and their potential influence on symptoms and clinical practice. Additionally, overlap with non-Covid autonomic dysfunction is considered. Understanding these new disorders can inform both neuro-immunology and Long Covid management.

## Long Covid—the new pandemic

Over 2% of the UK population now reports persistent symptoms following Covid infection [19], and post-Covid syndrome (or ‘Long Covid’) threatens physical health, workforce strength and societal wellbeing. The term ‘Long Covid’ encompasses ongoing symptomatic Covid (from 4 to 12 weeks) and post-Covid syndrome (symptoms lasting >12 weeks, not caused by an alternative diagnosis) [20]. Post-Covid syndrome is likely to be an ‘umbrella’ term for a wide range of undetermined conditions and physiological states. The definition from National Institute of Clinical Excellence emphasizes the heterogeneous multi-system nature of the syndrome, which can manifest as symptom clusters, often fluctuating over time [20]. Ambiguity about the condition (and lack of treatment options to date) can cause considerable uncertainty and anxiety for both clinicians and patients. An emerging but incompletely understood phenomenon increasingly seen in Long Covid and syncope clinics is cardiovascular autonomic dysfunction including postural orthostatic tachycardia syndrome (POTS) and orthostatic hypotension. Study of this apparent association with Long Covid may reveal underlying mechanisms and offer insight into non-Covid autonomic disorders with comprehension of autonomic nervous and neuroimmune system interaction.

Additionally, autoantibodies are increasingly being detected in Long Covid [18, 21–24]. Specifically, autoantibodies to inflammatory cytokines are present (such as IgG to IL-2, D8B, thyroglobulin and IFNδ) and these correlate with anti-SARS CoV2 IgG antibodies [25, 26]. Autoantibodies to antinuclear and extractable nuclear antigens are also elevated in individuals with Long Covid, and correlate with symptoms of fatigue and dyspnoea [18]. Notable autoantibodies include functionally active autoantibodies to the GPCRs (including α1- and β2-adrenoceptors, angiotensin receptor, nociception-like opioid receptor and muscarinic M2-receptor) [11]. This is significant for the overlap with POTS [12] and autonomic nervous system. Finally, the presence of GPCR autoantibodies in individuals with Long Covid correlates with impaired peripapillary vessel density in the eye, a biomarker for microcirculation health [27]. This partly validates theories of endothelial dysfunction and impaired coagulation.

A deep multi-omic investigation showed that some patients with Long Covid have autoantibodies at diagnosis, suggesting underlying subclinical autoimmunity [28]. Additionally, there are correlations with SARS-CoV-2 antibodies and symptoms of Long Covid, as well as a negative correlation between anti-SARS-CoV-2 IgG antibodies and autoantibodies [28].

Pro-inflammatory cytokines are associated with increased sympathetic activity which is also seen in non-Covid POTS [29]. This pro-inflammatory environment can stimulate microbiome-mediated autoantibody production, causing chronic immune activation or dysfunction [30]. Linking the above conditions are the common and non-specific symptoms of fatigue, cognitive symptoms and mental fatigue (so-called ‘brain fog’), gastrointestinal dysfunction and pain which are not unique to these diseases, but are non-specific sequelae of any immune dysregulation [31]. This is relevant in approach to therapeutics, which can either focus on underlying aetiology or consequential symptoms.

### Orthostatic intolerance syndromes—and what Long Covid can tell us about non-Covid POTS

These non-specific immune symptoms described above often accompany orthostatic intolerance symptoms—such as dizziness, light-headedness, palpitations, chest pain and, also reduced exercise tolerance. Such conditions include POTS (defined as orthostatic symptoms, a sustained heart rate rise of >30 bpm [or >40 bpm in individuals under 19 years of age]) on standing, without a reduction in blood pressure [32]. Orthostatic intolerance refers to symptoms and related haemodynamic changes when upright.

## Autonomic dysfunction in acute and Long Covid

### Long Covid autonomic dysfunction

Autonomic dysfunction presents as an important but incompletely understood component of Long Covid. There have been many reports of clinical cases and series describing POTS, inappropriate sinus tachycardia and orthostatic hypotension (all impairments of the cardiovascular autonomic nervous system) following Covid infection [33–42]. Vasovagal syncope has also been described [37, 43], but it is not yet clear that this is due solely to the viral infection given its frequency in the general population. Indeed, this applies to all cardiovascular autonomic conditions given the lack of detailed autonomic data in this population prior to the onset of Long Covid.

The assessment of cardiovascular autonomic integrity involves some important tests and markers of autonomic dysfunction such as tilt testing, active standing test, Valsalva manoeuvre, deep breathing test, Holter ECG and 24-h ambulatory blood pressure monitoring [44]. Impaired autonomic function is seen to be an adverse prognostic marker in both acute and Long Covid. Heart rate variability (HRV) is the variation in time between successive heartbeats, and is a biomarker of autonomic health. It is usually derived from 24-h Holter ECG or shorter ECG registration [45], and is reduced following Covid infection [46, 47]. Even resting tachycardia during acute infection (a sign of sympathetic activity) is associated with a more severe disease course [48], independent of comorbidity and fever [49]. Increased HRV predicts survival in people with acute Covid over 70 years, and lower HRV increases risk of admission to critical care [50]. In young adults recovering from Covid, elevated sympathetic nervous system (SNS) activity is apparent despite normal heart rate and blood pressure readings, with exaggerated responses on standing [51, 52]. In survivors at 6 months following hospital discharge, HRV parameters correlate inversely with pulmonary fibrosis and diffusion restriction [53]. One group of post-Covid patients had higher sympathetic activity and lower parasympathetic activity than controls, but these changes were more marked in individuals who were obese or physically sedentary [52]. These individuals were not symptomatic, and so the direct clinical implications are less clear, but the findings of many of the studies reported above are nevertheless compelling as they highlight the dynamic nature of HRV and potential for modulation.

## Mechanisms of orthostatic intolerance

**Figure 1.**
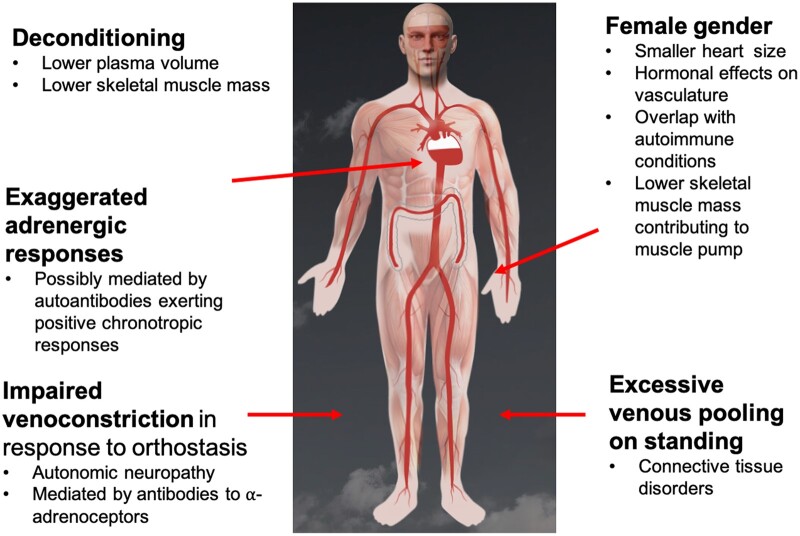
Possible mechanisms contributing to POTS. The underlying mechanisms are likely related to excessive venous pooling on orthostasis which is caused by excessive venous distension. In response to this reduction in cardiac venous return, increased SNS output occurs, increasing venoconstriction which may be impaired if small fibre neuropathy is present. Additionally, autoantibody-mediated impairment of peripheral vasoconstriction may occur. Tachycardia occurs in response to reduced venous return, in order to maintain cardiac output. Deconditioning can result in smaller left ventricular mass, requiring higher heart rate to maintain cardiac output. Possible reasons for the higher prevalence in women could be smaller heart mass, smaller muscle pump and an overlap with autoimmunity. Figure adapted with permission from www.stopfainting.com

These symptoms and responses relate to the individual’s responses to venous pooling and subsequent haemodynamic responses (Fig. 1). In brief, when a healthy individual stands up, up to a third of their blood volume pools in their lower body vasculature (in the pelvis and legs) by gravitational forces [54]. The consequent reduction in cardiac venous return is recognized by stretch receptors in the carotid sinus and aortic arch, and mechanoceptors in the heart and lungs. These signals are processed in the brainstem via afferent fibres, and increased sympathetic noradrenergic output occurs via efferent sympathetic neurons. These result in vasoconstriction of lower body vasculature, and increased heart rate and inotropy to maintain cardiac output in healthy individuals. Simultaneously, the renin angiotensin system is activated and anti-diuretic hormone (ADH) is secreted, promoting volume expansion and water retention. However, these responses may be impaired in those with orthostatic intolerance syndromes who may have inadequate blood volume, excessive venous pooling in the lower body or impaired peripheral vasoconstriction (e.g. by antibodies to the adrenergic receptors in lower limb blood vessels) [33]. The elevated compensatory adrenergic and noradrenergic responses occur daily, extending into weeks and months, and portend chronic multi-system constitutional symptoms. The symptoms of venous pooling and reduced cardiac output may be dizziness, fatigue, weakness and these changes are considered to be important in the evolution of vasovagal syncope [55].

## POTS ‘plus’ symptoms—and a link with constitutional immune symptoms

It is increasingly recognized that these haemodynamic abnormalities can be accompanied by multi-system constitutional symptoms which may have a major impact on symptom burden [56, 57]. Patients with POTS have a 5-fold higher symptom severity [58] than those without POTS—and while palpitations may be expected, less specific symptoms of fatigue and concentration difficulties are also prominent [58]. The activity restriction and morbidity associated with orthostatic intolerance syndromes is debilitating, and the non-specific fatigue, post-exertional malaise and cognitive symptoms (‘brain fog’) can be disproportionate to both the initial illness and the haemodynamic findings. Furthermore, those affected most commonly are in young and middle age, when they often have work and caring responsibilities, substantially reducing quality of life, sense of identity and employment.

While the association between these multi-system symptoms and POTS is notable, it is important to accept that there is no conclusive aetiological link identified. Ascribing such symptoms without a clear haemodynamic correlate carries risk of misdiagnosis, or worse, missing a potentially treatable other condition which mimics POTS [59]. This again highlights the need for a systematic clinical approach, and the need for defined research populations.

## Support for an autoimmune mechanism in POTS and other forms of stress

There is a compelling association between POTS and autoimmunity: POTS is associated with a higher-than-expected frequency of defined autoimmune disorders. One cohort had positive anti-nuclear antibodies in 25% patients and a concurrent autoimmune diagnosis in 20% [43]. In a UK study, 4% of POTS patients had biopsy-confirmed coeliac disease, compared with 1% of controls, and 42% self-reported gluten sensitivity, compared with 19% of controls [61]. In a case series of 13 patients with confirmed Sjögrens Syndrome (SS), 8 fulfilled criteria for POTS [62] suggesting that if sought, other autoimmune conditions may be found to co-exist with POTS. As such, clinical suspicion and careful evaluation and work up are required. There is also a striking preponderance for POTS in women, with female:male ratios of 4–5:1 [63]. Potential mechanisms are the hormonal effects on vasculature, differences in heart and skeletal muscle size or the fact that oestrogen exposure and concomitant infection can synergistically trigger autoimmunity [60, 64]. Finally, inflammatory, immune and neuroendocrine protein biomarkers are altered in individuals with POTS compared with controls [65–67].

The response to standing in POTS is a form of orthostatic stress. However, other forms of stress can also increase autoimmunity risk: a large population- and sibling-matched retrospective cohort study in Sweden found that exposure to life stressors confers a 36% increased risk of autoimmune disease [68]. If the stress-related disorder is post-traumatic stress disorder, the risk is even higher [68]. As such, the underlying stressors of Covid infection, Long Covid and orthostatic stressors (along with psychosocial stressors related to chronic illness) may confound an association between POTS and autoimmunity. A further factor is the detrimental effect of stress in other immune-mediated conditions, such as cancer [69] and atherosclerosis [70], suggesting that it may be a common underlying factor in development of a wide range of diseases. There are no data examining pre-morbid levels of anxiety or psychological factors prior to onset of Long Covid and POTS but prevalence of anxiety and depression are high in these conditions [71, 72]. This includes altered emotional responsivity during orthostatic stress and increased vigilance towards symptoms [73, 74].

## Auto-antibodies in POTS and Long Covid—significance and implications for future study

The association between POTS and autoantibodies is well recognized: auto-antibodies to the α and β receptors on GPCR antibodies, ganglionic acetylcholine receptor antibodies, angiotensin II receptor antibodies and antibodies to structural cardiac proteins have been identified in POTS [14, 27, 75–78]. The presence of autoantibodies can correlate with symptoms such as gastrointestinal disturbance [79], fatigue, muscle pain [80], exercise tolerance and standing time [81]. Antibodies to the α- and β- adrenoceptors may exert effects in POTS by either impaired vasoconstriction (mediated by α-adrenoceptors in the peripheral vasculature) resulting in venous pooling and reduced cardiac venous return) or positive chronotropic effects, exacerbating postural heart rate rise [11].

However, autoantibodies against GPCRs are not novel and they are described across a wide state of conditions in diseases ranging from SS to peripartum cardiomyopathy [82]. Specifically, GPCR antibodies have been found in disorders commonly associated with POTS, including inappropriate sinus tachycardia [83], complex regional pain syndrome [84] and chronic fatigue syndrome [85].

Although there is no causative link currently identified, the presence of autoantibodies is associated with a dysregulation of the immune response and, also, activation of transcription factors that may increase inflammatory responses. It is likely that more will be discovered in relation to both POTS and Long Covid. While the association between POTS and autoimmunity is compelling, understanding of specific mechanisms remains limited and as with many of the autoantibodies above, the specific mechanisms by which these autoantibodies exert effects, and their overall mechanisms, remain unclear. While their presence may reflect an underlying autoimmunity, it is essential to distinguish between pathogenic antibodies, and those antibodies suggesting mere autoimmunity but without a specific pathophysiological effect. This is an essential step in future research before treatment targets are developed.

There is also a need for standardization of antibody detection techniques. Currently, enzyme-linked immunosorbent assay (ELISA) is mostly used to detect human autoantibodies; however, non-specific binding is a well-recognized problem leading to frequent false positive results [86]. In a recent study, there was no difference in concentration of circulating antibodies against the most common cardiovascular GPCRs between POTS patients and controls using standard ELISA methodology [87]. This observation questions the use of ELISA for future explorative studies on POTS and Long Covid-associated dysautonomias and more studies are required for definitive conclusions. Non-specific binding sera contain increased concentrations of IgG and other inflammatory mediators, suggesting that this non-specific finding correlates with the IgG concentration and is therefore indicative of elevated IgG and inflammation [86].

Immunoprecipitation is also commonly used, but this has been difficult to develop for GPCRs. Equally, the requirement for radioisotopes limits its widespread application [88].

To summarize, multiple autoantibodies are identified in relation to both Long Covid and POTS, suggesting a possible link with autoimmunity, but the significance of these is as yet undetermined.

## Associations with mast cell activation disorders

A prominent overlapping symptom cluster associated with both POTS [56, 57] and Long Covid [89, 90] includes a multi-system and often non-specific symptom cluster which is often referred to as ‘mast cell activation syndrome’ (and considered to be due to release of mast cell mediators) and includes gastrointestinal disturbances (nausea, vomiting, diarrhoea, cramps), asthma like symptoms, flushing, urticaria, angioedema and constitutional symptoms such as fatigue and fever [91]. Mast cells are found in multiple tissues across a wide range of organs, and are critical in neuro-immune responses, particularly to allergy and inflammation. They contain multiple key pro-inflammatory cytokines as well as allergic and inflammatory mediators such as histamine, heparin and tryptase, which are released on degranulation. Disorders can range from mast cell leukaemia and systemic mastocytosis (aggressive, life-limiting conditions) to the less-specific, more benign and much more common ‘idiopathic MCAS’ where symptoms of mediator release are present, and biochemically confirmed, but without a confirmed mutational or pathological underlying cause [91].

Rigorous characterization studies relating to the relationship between POTS and MCAS are lacking; moreover, there is still controversy around specific criteria for defining mast cell disorders. Different diagnostic consensus criteria exist, which are more [92, 93] and less [91, 94] restrictive. Criteria range from differentiating mutational, pathological or biochemical biomarkers, to more non-specific syndromes characterized by symptom clusters which can be attributed to other conditions. An argument for precise, biomarker-led diagnoses (comprising bone marrow mast cell morphology, mutational analysis and plasma and urinary mast cell mediator levels) ensures precise diagnosis and avoids overdiagnosis of non-specific symptoms clusters and missing important alternative diagnoses, but similarly misses an opportunity to treat empirically a common and debilitating condition [94].

In any case, the prominent co-existence of these symptoms in those with Long Covid and orthostatic intolerance syndromes is notable and should be the focus of rigorous, biomarker-led characterization studies. There is little convincing evidence to date. However, Shibao *et al*. [95] tested the hypothesis that plasma histamine can cause sympathetic overactivity via vasodilatation and compared POTS patients presenting flushing and raised urinary methylhistamine (a mast cell mediator metabolite) with patients reporting flushing but with normal urinary metabolites. These MCA/POTS patients had predominant flushing breathlessness, headache, lightheadedness, excessive diuresis, diarrhoea, nausea and vomiting, and had a marked hyperadrenergic orthostatic response, with higher noradrenaline levels and exaggerated blood pressure rise on standing compared with the POTS and control groups [95]. In another study of 69 patients with POTS, 64% reported additional non-orthostatic symptoms such as allergic complaints, skin rash or symptoms, and two-thirds of these had elevated prostaglandins and elevated histamine markers [57]. An observational study of individuals recovering from Long Covid found that 72% individuals who received histamine antagonists reported a symptomatic improvement [96]—however, placebo effects must be considered. Finally, emphasis on the lack of generally accepted and available as well as sensitive, and reliable tests for mast cell activation disorders is necessary, which hampers the explorative studies and comparisons between different patient cohorts.

## An approach to management

Managing both Long Covid and POTS requires a multi-disciplinary holistic approach to understanding the wide-ranging symptoms and debilitating effects on daily life. A systematic multi-system approach, incorporating education and patient empowerment, as well as acknowledging the uncertainty around pathophysiology and the lack of evidence-based treatments, should be at the centre of the consultation, along with a tailored management plan incorporating established approaches and symptomatic treatments. This is summarized in Table 1.

**Table 1.** General approach to management of orthostatic intolerance syndromes, POTS and Long Covid POTS

Principle	Example
Multi-disciplinary team management	Primary and secondary care clinician, specialist nurse, psychologist, physiotherapist, occupational therapist, social worker, social prescriber
Patient education and health promotion	Patient support websites and apps Social prescribing Gentle paced exercise retraining groups Enabling self-management at home using apps, blood pressure monitoring and symptom diaries Setting expectations for living with chronic disease
Holistic management incorporating principles of integrative care	Support groups encouraging peer support and a sense of community Breath retraining Structured rehabilitation programmes
Reducing lower body venous pooling	Lifestyle: fluid and salt repletion, compression garments, isometric counter-pressure manoeuvres Pharmacotherapy: fluid expanders (e.g. fludrocortisone) and α-receptor agonists (e.g. midodrine) to promote splanchnic vasoconstriction
Managing reactive adrenergic response to orthostatic intolerance	Lifestyle: breath retraining exercises, meditation Pharmacotherapy: β-receptor blockers, ivabradine
Symptomatic management of symptoms suggestive of mast cell activation	H1-receptor and H2-receptor antagonists Mast cell stabilizing treatment

Managing orthostatic intolerance centres around reducing lower body venous pooling (expanding plasma volume, compression garments in lower body, especially lower abdomen, and isometric counter-pressure manoeuvres, α-agonist pharmacotherapy) [33], attenuating the reactive adrenergic response (e.g. β-blockers, breathing retraining), and slow cardiovascular and lower body reconditioning programmes, along with strong social and psychological support networks to facilitate managing a new and chronic condition. Pharmacotherapy may be needed to attenuate symptoms. However, there is insufficient evidence of underlying immune targets to consider immuno-therapeutics.

There have been case reports [97] and small open-label clinical studies [98] suggesting a beneficial effect of intravenous immunoglobulin therapy in POTS. While there are no controlled clinical trial data examining this, nor an established target to treat, it again supports a possible role for autoimmunity. However, as described above, the lack of distinct treatment targets and the specific role of autoantibodies being defined suggest that identifying an immunological target will take time to achieve [99].

Finally, in practice, we cautiously consider a trial of mast cell stabilizing medications for symptomatic benefit in patients significantly debilitated by symptoms—including H1 and H2 receptor antagonists and mast cell stabilizing treatment. Obtaining biomarker-led diagnoses including serum and urine metabolites is important, but not always pragmatic in an era of high volume, remote clinics, and we advocate a trial of such medications for a month if clinical suspicion is high. Our experience is that this approach is particularly helpful for individuals with nocturnal symptoms (including palpitations, shortness of breath and dizziness); a possible explanation is spontaneous release of mast cell mediators causing peripheral vasodilation and a reactive adrenergic response. Caution is required to balance benefits of symptomatic treatment with anticholinergic side effects, which can be harmful in older individuals or those with neurodegenerative disorders. Importantly, as before, placebo-effects must also be borne in mind.

## Conclusion

The neuro-immune and autonomic nervous systems are increasingly recognized in autonomic dysfunction in Long Covid and cardiovascular autonomic disorders such as POTS. Studies are still in their infancy but as evidence accrues, further links may become apparent. The common associations with autoantibodies are compelling, but non-specific, and antibody tests are not entirely reliable and available. Similarly, many symptoms of immune dysregulation may accompany these disorders and reflect a final common pathway rather than focal or specific pathology—and clinically, these need to be managed pragmatically and sympathetically. Cardiovascular dysautonomic conditions are debilitating and the effects on society are significant. The widespread incidence and prevalence of Long Covid has highlighted the knowledge gaps in POTS research and a dual approach for answers is urgently needed.

## Data availability 

No data available.

## Funding

No funding was required for this work.
